# The role of the microcirculation and integrative cardiovascular physiology in the pathogenesis of ICU-acquired weakness

**DOI:** 10.3389/fphys.2023.1170429

**Published:** 2023-05-10

**Authors:** Asher A. Mendelson, Dustin Erickson, Rodrigo Villar

**Affiliations:** ^1^ Section of Critical Care Medicine, Department of Medicine, Rady Faculty of Health Sciences, University of Manitoba, Winnipeg, MB, Canada; ^2^ Faculty of Kinesiology and Recreation Management, University of Manitoba, Winnipeg, MB, Canada

**Keywords:** ICU-acquired weakness (ICU-AW), critical illness, microcirculation, exercise physiology, oxygen delivery and consumption

## Abstract

Skeletal muscle dysfunction after critical illness, defined as ICU-acquired weakness (ICU-AW), is a complex and multifactorial syndrome that contributes significantly to long-term morbidity and reduced quality of life for ICU survivors and caregivers. Historically, research in this field has focused on pathological changes within the muscle itself, without much consideration for their *in vivo* physiological environment. Skeletal muscle has the widest range of oxygen metabolism of any organ, and regulation of oxygen supply with tissue demand is a fundamental requirement for locomotion and muscle function. During exercise, this process is exquisitely controlled and coordinated by the cardiovascular, respiratory, and autonomic systems, and also within the skeletal muscle microcirculation and mitochondria as the terminal site of oxygen exchange and utilization. This review highlights the potential contribution of the microcirculation and integrative cardiovascular physiology to the pathogenesis of ICU-AW. An overview of skeletal muscle microvascular structure and function is provided, as well as our understanding of microvascular dysfunction during the acute phase of critical illness; whether microvascular dysfunction persists after ICU discharge is currently not known. Molecular mechanisms that regulate crosstalk between endothelial cells and myocytes are discussed, including the role of the microcirculation in skeletal muscle atrophy, oxidative stress, and satellite cell biology. The concept of integrated control of oxygen delivery and utilization during exercise is introduced, with evidence of physiological dysfunction throughout the oxygen delivery pathway - from mouth to mitochondria - causing reduced exercise capacity in patients with chronic disease (e.g., heart failure, COPD). We suggest that objective and perceived weakness after critical illness represents a physiological failure of oxygen supply-demand matching - both globally throughout the body and locally within skeletal muscle. Lastly, we highlight the value of standardized cardiopulmonary exercise testing protocols for evaluating fitness in ICU survivors, and the application of near-infrared spectroscopy for directly measuring skeletal muscle oxygenation, representing potential advancements in ICU-AW research and rehabilitation.

## Introduction

Millions of patients are admitted annually to the intensive care unit (ICU) (Adhikari et al., 2010), and enhanced recovery in ICU survivors is recognized as a top priority for clinicians and researchers (Azoulay et al., 2017; Prescott et al., 2019). Intensive care unit-acquired weakness (ICU-AW) is a common disorder that can develop in critically ill patients, and is characterized by skeletal muscle breakdown, loss of strength/contractility, and impaired capacity for muscle regeneration and repair (Fan et al., 2014; Lad et al., 2020; Vanhorebeek et al., 2020). Patients with ICU-AW, compared with ICU patients that do not develop this condition, experience increased ICU length of stay and mortality (Sharshar et al., 2009; Moisey et al., 2013), increased 1-year mortality, and reduced functional independence and quality of life (Kress and Hall, 2014; Herridge et al., 2016). The reported incidence of ICU-AW is variable, partially due to inconsistent diagnostic criteria, ranging from 7% to 76% of ICU patients, with an approximate estimate of 40% in the general ICU population (Appleton et al., 2015). Baseline risk factors that increase the likelihood of developing ICU-AW are age (Moisey et al., 2013), and medical comorbidities such as chronic heart failure, anemia, and renal disease (Rudra et al., 2021); risk factors that develop within the ICU are duration of mechanical ventilation, diagnosis of sepsis, severity of critical illness, hyperglycemia, use of glucocorticoids and neuromuscular blockade, and prolonged immobilization (Yang et al., 2018). There are currently no targeted therapies that can prevent or reverse the natural trajectory of ICU-AW, signifying an urgent and unmet need in the treatment of this condition.

The biological mechanisms underlying ICU-AW are complex, commonly identified as increased protein catabolism, abnormal calcium signaling, transcriptional and post-transcriptional modification, mitochondrial dysfunction, and impaired satellite cell homeostasis–the reader is directed to recent excellent reviews for more details (Friedrich et al., 2015; Lad et al., 2020; Vanhorebeek et al., 2020; Wang et al., 2020). The common paradigm for studying ICU-AW focuses predominantly on the pathological changes within myocytes (i.e., intracellular) and associated motor neurons (Stevens et al., 2009), without sufficient consideration for their *in vivo* physiological environment. Particularly, the role of skeletal muscle microcirculation and microvascular milieu in initiating, propagating, and sustaining ICU-AW has not been studied. Moreover, the cardiovascular control of locomotion - through matching of global and regional oxygen supply with tissue demand - has rarely been explored within the context of ICU-AW. Intact and well-regulated cardiovascular and microvascular systems are a fundamental requirements for normal skeletal muscle function, and their dysfunction contributes to weakness and impaired exercise capacity in numerous disease conditions.

We believe these updated perspectives may provide novel insights into ICU-AW pathogenesis and opportunities for treatment. The aim of this review is to overview the significance of the microcirculation and integrative cardiovascular physiology in skeletal muscle health and disease, provide supporting evidence for their role in the pathogenesis ICU-AW using comparisons with other disease states, and bring forward unanswered questions and hypotheses for studying the microcirculation and cardiovascular system within the context of ICU-AW.

## Skeletal muscle microcirculation ‐ structure and function

The microcirculation defines the portion of the cardiovascular system comprising the smallest blood vessels throughout the body. Although there is no exact size cutoff, it is generally accepted that the microcirculation denotes vessels with internal diameter less than 100–150 microns, with upper estimates of 300 microns (Pries et al., 1996). Because of the direct interface with all organs, the microcirculation is tasked with the fundamental homeostatic roles of the cardiovascular system, including coagulation, nutrient delivery and waste removal, permeability, and immune signaling. We can surmise that chief among these roles is the regulation of oxygen delivery to meet local tissue demand, which is essential to maintain cellular respiration and support life. This is achieved by convective transport (i.e., blood flow) within the circulatory system over large distances, and diffusive transport from the microcirculation into surrounding tissue (Poole et al., 2022).

Microvascular networks share commonality throughout the body, but also manifest organ-specific structure and function to serve their specialized needs (e.g., liver, brain, lung). Skeletal muscle is the largest organ by mass, accounting for approximately 40% of total body weight (Davidson et al., 2014; Kim et al., 2016) and largest microvascular network (Poole et al., 2011). Due to its specialized function in locomotion, skeletal muscle also has the widest range of metabolic requirements, whereby oxygen consumption can increase 100-fold from rest to peak exercise (Poole et al., 2022). This increase in oxygen consumption and muscle work correlates with a proportional increase in muscle blood flow (Poole et al., 2011; Murrant and Sarelius, 2015), indicating exquisite mechanisms for matching oxygen demand with supply.

Skeletal muscle microvascular networks can be separated into two anatomical and functional domains: arterioles/venules, and capillaries. Arterioles are organized into branching trees and looping arcades within skeletal muscle (Engelson et al., 1986; Schmid-Schönbein et al., 1987; Williams and Segal, 1992) and accompanied by a paired venular system that follows the same branching architecture (Engelson et al., 1985; Pries et al., 1995). Skeletal muscle capillaries are located between muscle fibres (i.e., within the endomysium), and are organized into groups of 10–20 parallel capillaries originating from a terminal arteriole and draining into a post-capillary venule (Skalak and Schmid-Schönbein, 1986; Lund et al., 1987; Delashaw and Duling, 1988; Emerson and Segal, 1997). These groups are called capillary modules that form interconnected columns spanning thousands of microns (Tyml et al., 1981; Skalak and Schmid-Schönbein, 1986). Recently this columnar ultrastructure has been denoted as a Capillary Fascicle that is now recognized to be associated with the muscle fascicle (Mendelson et al., 2021).

How do these microvascular domains regulate blood flow and oxygen delivery in skeletal muscle? Arterioles are surrounded by circumferential smooth muscle, and can modulate their diameter and resistance in specific segments to distribute blood flow within large portions of the microvascular network (Segal, 2005). Myogenic tone provides a feedback loop, such that increases to intraluminal pressure will lead to vasoconstriction of the arteriolar smooth muscle (Davis, 2012); shear-dependent vasodilation, mediated by nitric oxide, provides an opposing mechanism that prevents significant biophysical stress associated with increased blood flow through resistance vessels (Gutterman et al., 2016). These signals are integrated along the arteriolar tree through sympathetic innervation of vascular smooth muscle (Thomas and Segal, 2004) and conducted vasodilation of depolarizing signals that travel *via* the vascular endothelium (Bagher and Segal, 2011) in order to match blood flow with local tissue metabolic demand. Capillary networks are the principal site of oxygen exchange in the microcirculation, and thus play a pivotal role in supporting healthy skeletal muscle function (Murrant et al., 2017; Poole, 2019). Capillaries, by contrast, lack smooth muscle, but are associated with mural cells called pericytes (Murray et al., 2017); however, the concepts of capillary recruitment and pre-capillary sphincters as loci of blood flow control have largely been abandoned (Poole, 2019). Although it was originally postulated that capillaries themselves were simply passive conduit vessels, empirical evidence suggests they are active participants in microvascular flow regulation, mediated through conducted signaling with upstream arterioles (Dietrich and Tyml, 1992; Cohen and Sarelius, 2002; Twynstra et al., 2012). Other mechanisms for microvascular signaling include oxygen-dependent ATP release from red blood cells (RBCs), triggering a depolarization event on the capillary endothelium (Ellis et al., 2012; Ellsworth et al., 2016).

## Microvascular dysfunction in critical illness–looking beyond the ICU

The microcirculation is now recognized as a central factor in the pathogenesis of critical illness (De Backer et al., 2011; 2014; Moore et al., 2015; Østergaard et al., 2015). This has been described most extensively in the context of sepsis - the dysregulated host response to infection that causes life-threatening organ dysfunction (Singer et al., 2016) - but is also applicable to other common ICU diseases including trauma, cardiac arrest, and following major surgery. Because of the diffuse systemic inflammatory response that characterizes the early host response of sepsis, microvascular dysfunction is ubiquitous, and is considered a cardinal feature of the disease (Spronk et al., 2004; Ellis et al., 2005; Ince, 2005).

Microvascular dysfunction in sepsis begins with activation of the endothelium, and change of endothelial cells (ECs) to a pro-inflammatory phenotype (Ince et al., 2016; Joffre et al., 2020). Pathogens, bacterial toxins, and bacterial degradation products directly activate endothelial cell-surface receptors that recognize pathogen-associated molecular patterns (PAMPs), as well as circulating damage-associated molecular patterns (DAMPs) released from injured human cells (van der Poll and Opal, 2008; Raymond et al., 2017). This EC pro-inflammatory phenotype results in shedding of the endothelial glycocalyx (Uchimido et al., 2019), disrupted endothelial barrier function, and increased vascular permeability (Opal and van der Poll, 2015; Hotchkiss et al., 2016). Activated ECs also have pro-coagulopathic and anti-fibrinolytic phenotype, causing adhesion of fibrin, complement factors, and platelets, eventually leading to widespread microthrombosis, disseminated intravascular coagulation, and organ injury (Stearns-Kurosawa et al., 2011; de Stoppelaar et al., 2014; Semeraro et al., 2015; Bermejo-Martin et al., 2018; Chang, 2019). This microvascular coagulopathy is exacerbated by leukocyte activation and secretion of neutrophil extracellular traps (NETs), consisting of cell-free DNA and anti-microbial enzymes to combat pathogens that are easily incorporated into fibrin microthrombi (Denning et al., 2019).

From a hemodynamic perspective, endothelial activation causes vasodilation through upregulation of inducible nitric oxide synthase (iNOS) within ECs acting on arteriolar smooth muscle (Bateman et al., 2003), which contributes to the observed hypotension in sepsis. Increase in nitric oxide production occurs heterogeneously throughout the microcirculation causing inappropriate shunting of blood flow and oxygen through microvascular networks (Miranda et al., 2016). Human studies in septic patients assessing hyperemic responses of the brachial and femoral arteries (as markers of nitric oxide-dependent vascular function) also suggest that nitric oxide bioavailability is attenuated (Nelson et al., 2016); this may reflect differential physiology of upregulated iNOS and downregulated endothelial nitric oxide synthase (eNOS) pathways (Lambden, 2019). Moreover, arterioles demonstrate decreased adrenergic sensitivity and hypo-responsiveness to catecholamines that exacerbates sepsis-induced hypotension (Kimmoun et al., 2013; Burgdorff et al., 2018). Sepsis also has an effect on blood flow throughout the microcirculation, causing a turbulent shear stress on ECs (Lupu et al., 2020) which exacerbates endothelial injury. At the level of the capillary network, sepsis causes microvascular alterations that impair oxygen transport. Reduced RBC velocity, RBC supply rate, and RBC oxygen saturation are routinely observed in skeletal muscle capillary networks (Ellis et al., 2002; Bateman et al., 2008; Kowalewska et al., 2022), signifying impaired transport of oxygen. Stopped-flow capillaries are considered a hallmark feature of sepsis (Lam et al., 1994; Piper et al., 1996; Ellis et al., 2002; Bateman et al., 2008); consequently, oxygen diffusion capacity of capillary networks is reduced which impairs oxygen delivery to surrounding tissue. Breakdown of intercellular gap junctions impairs conducted signaling from capillaries to upstream arterioles (Lidington et al., 2003), which limits communication and blood flow regulation throughout the microvascular network. Furthermore, sepsis reduces the ability for RBCs to release ATP and to signal appropriately in the setting of RBC hypoxia (Bateman et al., 2015), and decreases RBC deformability which contributes to RBC maldistribution in capillary networks (Bateman et al., 2017).

Human studies have also demonstrated microvascular dysfunction during the acute phase of critical illness using handheld vital microscopy (HVM) of the sublingual microcirculation, as well as skeletal muscle near-infrared spectroscopy (NIRS). De Backer and others were among the first to identify loss of microvascular perfusion in septic patients using sublingual HVM (De Backer et al., 2002), and further showed that proportion of perfused vessels (PPV)—analogous to stopped-flow capillaries observed in preclinical models - was an independent predictor of ICU mortality (De Backer et al., 2013). HVM-derived indices of microvascular perfusion were also noted by Hernandez to correlate with mortality, organ failure, lactate, and vasopressor requirements but not systemic hemodynamics (Hernandez et al., 2013). Similar microvascular dysfunction was noted during acute resuscitation in non-survivors of sepsis even though blood pressure and central venous oxygenation were normalized (Trzeciak et al., 2008). Edul and others showed that RBC velocity was reduced in patients with sepsis, and similar flow reductions were observed independent from measurements of adequate cardiac output (Kanoore Edul et al., 2016). In the ICU, skeletal muscle NIRS has been investigated for providing quantitative and reproducible measurements of the peripheral microcirculation, including skin, soft tissue, and underlying skeletal muscle (Mesquida et al., 2013; Možina and Podbregar, 2015); tissue oxygen saturation (StO2) with or without vascular occlusion test (VOT) is the most common metric derived from skeletal muscle NIRS. For patients with sepsis, numerous studies have demonstrated that abnormal StO2 measurements in skeletal muscle are negative prognostic markers for ICU outcomes (Skarda et al., 2007; Neto et al., 2014), which is observed even when systemic hemodynamics were normalized after initial resuscitation (Leone et al., 2009; Lima et al., 2009). Additionally, circulating biomarkers of endothelial injury have been associated with increased mortality in human sepsis (Shapiro et al., 2010; Skibsted et al., 2013; Hendrickson and Matthay, 2018; Lafon et al., 2020).

However, microvascular research in critical illness has focused almost exclusively on observations during ICU admission. Accordingly, the persistence of microvascular dysfunction during recovery from critical illness and its potential contribution to ICU-AW remains largely unknown. Sakr and others demonstrated that PPV was lower in non-survivors vs. survivors of sepsis throughout the duration of ICU admission (Sakr et al., 2004). De Backer found a general improvement in microvascular function over the course of ICU admission for all patients, but non-survivors were still worse compared to survivors when measured in the late phase of illness (>60 h) (De Backer et al., 2013). Donati and others used skeletal muscle NIRS and VOT for longitudinal measurements in ICU patients from admission until death or discharge (Donati et al., 2016). They found that while StO2 was comparable between survivors and non-survivors at discharge or death, the reoxygenation slope was worse for patients who died compared to those who survived. These few studies suggest that more research is needed to characterize the long-term natural trajectory of skeletal muscle microvascular dysfunction after ICU discharge in the recovery phase after critical illness.

## Endothelial-myocyte crosstalk and molecular mechanisms of ICU-AW

Intracellular events and molecular pathways within skeletal muscle cells (myocytes) that initiate and propagate ICU-AW have been well-described (Lad et al., 2020), but the contribution of the surrounding microcirculation remains largely unexplored. Endothelial cells (ECs) are the specialized cells that line blood vessels within the microcirculation, and are active participants in the physiology of their surrounding organ. EC crosstalk can be broadly defined and the biological or physiological communication between ECs and any other cell of interest. Notable examples include maintenance of the blood-brain-barrier (Sweeney et al., 2019) and gut vascular homeostasis (Spadoni et al., 2015), where endothelial permeability and immune signaling are essential for well-regulated function. Moreover, EC crosstalk has been described in numerous pathological conditions including inflammatory bowel disease (Cromer et al., 2011), diabetic and ischemic cardiomyopathy (Wan and Rodrigues, 2016; Colliva et al., 2020), neuroinflammation (Greenwood et al., 2011), adipocyte dysfunction in metabolic syndrome and obesity (Sabaratnam and Svenningsen, 2021), and acute respiratory distress syndrome (Millar et al., 2016). This crosstalk phenomena can be mediated through endothelial-secreted proteins, termed angiocrine signaling (Rafii et al., 2016), or through endothelial-derived extracellular vesicles that can have local or distant effects (Mathiesen et al., 2021).

EC-myocyte crosstalk is a topic of research with promising application for ICU-AW. Inflammatory activation of the endothelium is a recognized feature of critical illness (Joffre et al., 2020), and results in secretion of numerous mediators that may subsequently affect muscle function; indeed, inflammatory activation of skeletal muscle is a central theory regarding the acute phase of ICU-AW (Friedrich et al., 2015; Lad et al., 2020). Patients who develop ICU-AW have significantly higher levels of systemic inflammation compared to ICU patients do not develop ICU-AW (Witteveen et al., 2017), and chronic inflammatory conditions such as cancer are well-recognized to activate catabolic pathways in skeletal muscle, such as the ubiquitin proteasome system (Webster et al., 2020). EC secretion of IL-6 with subsequent action on skeletal muscle would be an obvious candidate, because it is an archetypal inflammatory molecule that secretion of which is well-described by ECs during sepsis (Joffre et al., 2020). Intramuscular injection of IL-6 in mice has been shown to elicit a significant decrease in skeletal muscle mass (Haddad et al., 2005), and exposure of C2C12 myotubules *in vitro* to exogenous IL-6 upregulated mitochondrial reactive oxygen species (ROS) with negative effects on cellular stress and mitochondrial respiration (Abid et al., 2020). Moreover, skeletal muscle-derived IL-6, commonly secreted during exercise (Muñoz-Cánoves et al., 2013), appears to play a role in systemic leukocyte trafficking and cytokine response in a preclinical model of polymicrobial abdominal sepsis (Laitano et al., 2021), suggesting a potential differential effect depending on the origin of the signaling molecule. ICU-AW studied with LPS activation of C2C12 myoblast cells *in vitro* is potentiated by TNF-alpha (Ono and Sakamoto, 2017) and adrenergic stimulation (Matsukawa et al., 2021)—both of which are upregulated in ECs during critical illness. Furthermore, the overlapping role of oxidative stress in EC dysfunction and skeletal muscle dysfunction (Huet et al., 2011; Powers et al., 2011) suggests that ROS may be an important locoregional effector that communicates between these cell types during ICU-AW. Overall, however, the specific molecules and pathways that mediate crosstalk between activated ECs and myocytes during inflammation and ICU-AW remain largely uncharacterized.

Another important feature of ICU-AW is impaired capacity for regeneration of skeletal muscle after injury and atrophy (Batt et al., 2019; Lad et al., 2020), and EC-myocyte crosstalk may play an important role here as well. Satellite cells are muscle-specific stem cells that proliferate differentiate, and fuse to give rise to myoblasts and myocytes (Yin et al., 2013). Disruption to these cells limits the rate at which muscle mass can be regained after catabolism, contributing to the long-term effects of ICU-AW. Muscle biopsies of patients with persistent skeletal muscle atrophy at 6-month after ICU discharge demonstrate reduced satellite cell count compared to patients with improved muscle mass (Dos Santos et al., 2016), and these findings are corroborated in animal models of sepsis-induced muscle atrophy (Rocheteau et al., 2015). Satellite cells are closely localized with adjacent capillaries in a “vascular niche” (Yin et al., 2013; Collins and Kardon, 2018), and myogenesis and angiogenesis are tightly coupled in regenerating muscle (Yin et al., 2013; Latroche et al., 2017) with bi-directional secretion of pro-angiogenic and pro-myogenic factors between both cell types (Christov et al., 2007). In response to VEGF secreted from satellite cells, ECs proliferate towards the satellite cells, decreasing the perfusion distance for surrounding muscle tissue (Verma et al., 2018); satellite cells have also been shown to respond to VEGF by inducing proliferation and differentiation into myotubes (Arsic et al., 2004). Lactate secreted from ECs appears to play a role in polarizing skeletal muscle macrophages towards M2 repair (Zhang et al., 2020). Notch signaling from ECs drives satellite cells into quiescence (Verma et al., 2018), and dysfunction in this pathway contributes to a rapid depletion in satellite cells in Duchenne muscular dystrophy (Jiang et al., 2014). It has also been shown that ECs cultured in a hyperglycemic medium secrete factors that impede satellite cell activation and differentiation (Kargl et al., 2019). Together, these studies support a compelling hypothesis that EC dysfunction and satellite cell dysfunction are co-dependent phenomena that sustain each other and limit skeletal muscle regeneration after critical illness.

**FIGURE 1:**
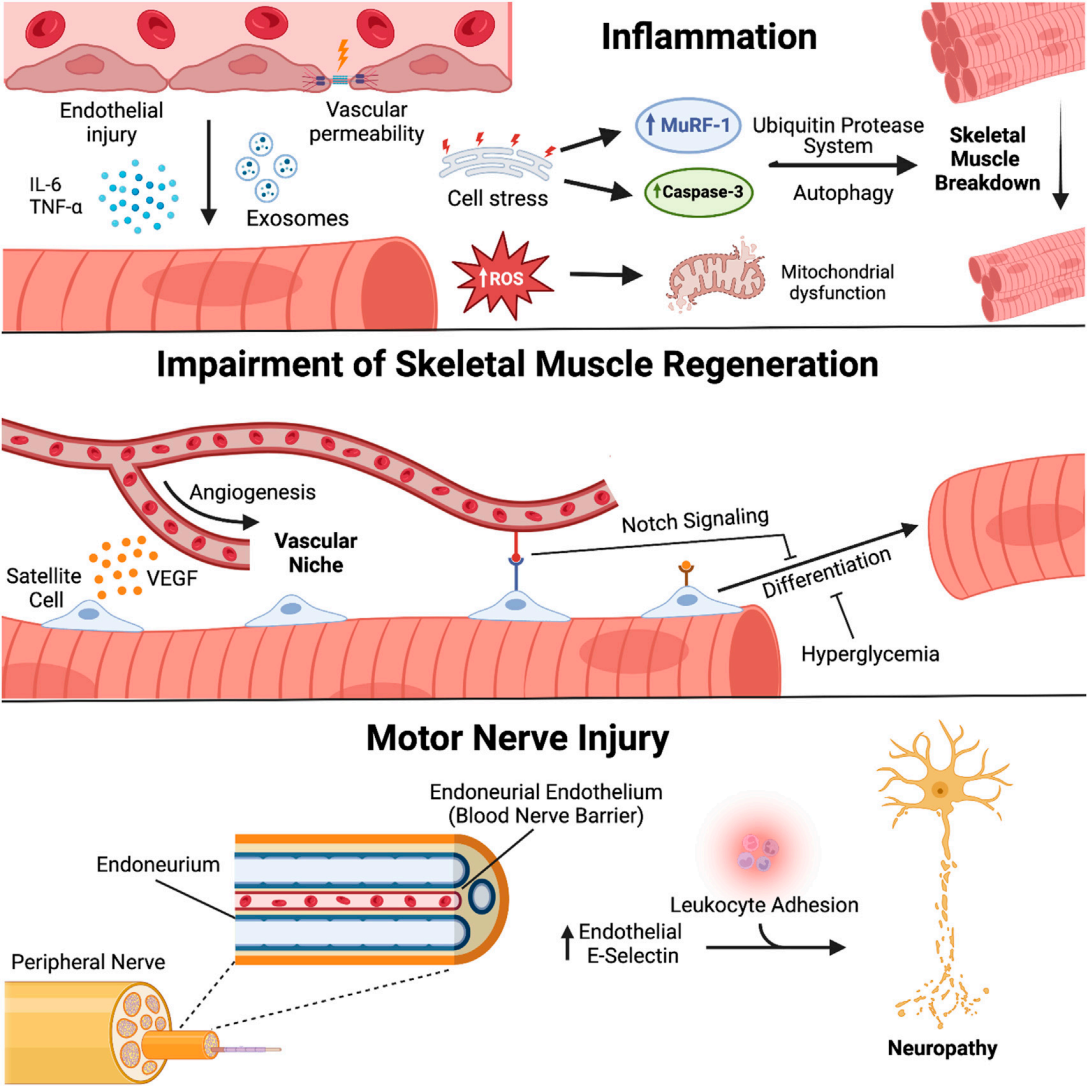
Endothelial biology and pathogenesis of ICU-acquired weakness. Endothelial cells (EC) engage in molecular crosstalk with myocytes and peripheral nerves in skeletal muscle. Activated ECs secrete mediators of inflammation that lead to atrophy and oxidative stress within adjacent myocytes; disruption of the vascular niche affects satellite cell function and capacity for muscle regeneration and repair; damage to the blood-nerve-barrier promotes leukocyte adhesion and development of critical illness polyneuropathy. *Created with* *BioRender.com* .

Contributions of the endothelium to ICU-AW also occur within the peripheral nervous system through development of critical illness polyneuropathy (CIP) (Batt et al., 2013). Similar to the blood brain barrier, ECs are involved in creating a blood nerve barrier (BNB), an area surrounding axons of the peripheral nervous system that is responsible for regulating the diffusion of molecules from the microcirculation (Mizisin and Weerasuriya, 2011). E-selectin, an EC surface molecule involved in the adhesion of leukocytes, was found to be upregulated on the endoneurial endothelium in peripheral nerve biopsies of critically ill patients (Fenzi et al., 2003). Recruitment of immune cells and their pro-inflammatory response will cause vascular permeability and tissue damage acting on the endoneurium and the peripheral nerves contained within, which is suggested to play a role in BNB disruption and axonal degeneration found in CIP (Fenzi et al., 2003). A summary of the mechanisms of endothelial biology and ICU-AW described in this section is found in Figure 1.

## Substrate delivery and impaired skeletal muscle function–role of the microcirculation

Another way that the skeletal muscle microcirculation may play a role in the pathogenesis of ICU-AW is through ineffective nutrient and metabolite transfer during the acute phase of critical illness, and also in the chronic phase in ICU survivors with established weakness. The two primary substrates required for adequate cellular respiration are oxygen and glucose; of these two, oxygen requires continuous resupply and cannot be stored, whereas skeletal muscle does have capacity to release glucose from glycogen (Bar-Or et al., 2019).

When microvascular systems lose ability to regulate blood flow and oxygen delivery, skeletal muscle dysfunction ensues due to lack of adequate energy supply. This has been studied in numerous chronic disease conditions outside of critical illness. Maladaptive arteriolar response to vasodilators contributes to skeletal muscle blood flow heterogeneity and hypoxia in preclinical models of metabolic syndrome (Frisbee et al., 2016; 2019). Impairments have also been described in diseased skeletal muscle capillary networks (Hirai et al., 2015; Poole, 2019; Poole et al., 2022)—with functional limitations appearing to play a larger role than structural changes (although both occur). Reduced capillary RBC flow and blunted capillary hyperemic response are features of impaired skeletal muscle oxygen delivery in preclinical models of heart failure (Kindig et al., 1999; Richardson et al., 2003) and aging (Copp et al., 2009). There is also significant correlation between reactive hyperemia-peripheral arterial tonometry index scores and hand grip strength in elderly patients (Yoo et al., 2018), suggesting that endothelial function is associated with the severity of sarcopenia and overall function of skeletal muscle.

Microvascular dysfunction in skeletal muscle is an early event in the pathogenesis of critical illness, and has been shown to occur in rodent models of sepsis within 3–4 h after administration of intraperitoneal fecal slurry (Ellis et al., 2002; Goldman et al., 2006; Kowalewska et al., 2022). These microvascular alterations manifest primarily as blood flow heterogeneity with both hyperemic and stopped-flow capillaries. Using computational modeling, Goldman and others demonstrated how flow heterogeneity in sepsis (particularly stopped-flow capillaries) accounts for much of the pathological supply dependency of oxygen that is encountered in skeletal muscle (Goldman et al., 2004; 2006). Accordingly, it has been shown that regions of hypoxia occur within the muscle are associated with microvascular dysfunction and increased metabolic demand during critical illness (Fraser et al., 2015). Acute skeletal muscle hypoxia appears to induce changes in mitochondrial biogenesis and ROS signaling via the NRF2 transcription factor (Ji et al., 2018), which has been implicated in ICU-AW (Kanova and Kohout, 2022); however, the role of acute hypoxia signaling pathways in the early pathogenesis of ICU-AW remains largely unexplored.

Chronic hypoxia and skeletal muscle dysfunction is well-established, but the potential connection with long-term ICU-AW is not yet clear. Prolonged hypoxia paired with a decrease in activity has been shown to increase the rate of breakdown in skeletal muscle independent of age (Debevec et al., 2018). Even without any changes to activity, prolonged exposure to hypoxic conditions has been shown to increase the levels of muscle ring finger protein 1 (MuRF-1) by almost two-fold (Chaillou et al., 2014), which is a ubiquitin protease implicated in the pathogenesis of muscle breakdown and ICU-AW (Constantin et al., 2011). Hypoxia also contributed to increased skeletal muscle myostatin expression in rats and humans, resulting in muscle atrophy (Hayot et al., 2011). Reduced skeletal muscle capillary density–which is an important determinant of oxygen diffusion capacity—has been associated with reduced exercise capacity in aging (Landers-Ramos and Prior, 2018), and in patients with peripheral arterial disease (Duscha et al., 2020) and congestive heart failure (Duscha et al., 1999); conversely, exercise training has been shown to improve skeletal muscle performance through a number of adaptive mechanisms, including increased capillary density (Gavin et al., 2007; Haas and Nwadozi, 2015). *Ex vivo* models of prolonged hypoxia in murine skeletal muscle cells demonstrated increased satellite cell differentiation, which the authors speculated was an adaptive mechanism to overcome limited oxygen availability (Elashry et al., 2022). Similarly, in human skeletal muscle biopsies in patients with COPD, increased markers of angiogenesis were observed (particularly angiopoietin-2), which were negatively correlated with lung function and positively correlated with muscle wasting (Mofarrahi et al., 2013). Collectively, slowed oxygen kinetics create an “oxygen debt” within contracting myocytes, leading to intracellular metabolic derangements in phosphocreatine (PCr), adenosine diphosphate (ADP), acidosis, and glycogenolysis (Poole et al., 2012; Hirai et al., 2015). Consequently, skeletal muscle hypoxia in disease results in a shift of muscle fibre type from slow-twitch (type 1, oxidative) to fast-twitch (type 2, glycolytic) (Drexler et al., 1992; Schaufelberger et al., 1997; Kitzman et al., 2014)—potentially signifying an adaptive strategy to accommodate reduced substrate delivery. This has also been observed with COPD, whereby patients have a significant decrease in oxygen consumption (VO_2_) as well as a decrease in the percentage of type I and an increase of type 2 fibres within the vastus lateralis (Maltais et al., 1999). Dos Santos and others observed a non-significant trend towards decreased capillary-to-myocyte ratio in atrophic quadriceps in ICU survivors at 6 months (Dos Santos et al., 2016). However, muscle biopsies from ICU patients typically reveal loss of both fibre types, but preferential loss of type 2 fibres (Bierbrauer et al., 2012; Wollersheim et al., 2014), suggesting that multiple factors beyond hypoxia may be implicated. The other explanation for these potentially discordant findings is that these biopsies occurred during the early phase of the disease, before the effects of chronic hypoxia could become established. In this regard, ICU-AW is recognized as a multi-phased syndrome with both early and late changes evolving in the muscle (Files et al., 2015).

Hyperglycemia is a classical stress response to acute critical illness, resulting from cellular, metabolic, and hormonal changes throughout the body (Marik and Bellomo, 2013; Bar-Or et al., 2019). Notably, hyperglycemia is recognized as a risk factor for the development of ICU-AW (Friedrich et al., 2015; Yang et al., 2018). Skeletal muscle is responsible for approximately 80% of the body’s glucose uptake in response to insulin (DeFronzo et al., 1985; Honka et al., 2018), and therefore consideration for glucose homeostasis in critical illness invariably requires attention to skeletal muscle. While it is recognized that sepsis impairs skeletal muscle glucose uptake via downregulation of skeletal muscle GLUT-4 transporter (Lombardi et al., 1999; Lu et al., 2015), the role of the microcirculation has recently been evaluated. Using a rodent model of fecal peritonitis, Magnemi and others show that impaired microvascular perfusion causes 50% reduction in insulin delivery to skeletal muscle (Mignemi et al., 2019). In this study, capillary insulin permeability was unaffected, suggesting that the reduction in insulin delivery is due to the disruption of microvascular bulk flow. Thus, while the deleterious cellular mechanisms attributed to systemic hyperglycemia (e.g., ROS) certainly contribute to acute myocyte toxicity and initiation ICU-AW, it may also be true that hyperglycemia is a surrogate for skeletal muscle microvascular dysfunction.

## Integrative cardiovascular physiology–insights into weakness from a systems perspective

Regulation of oxygen (O_2_) delivery to match O_2_ utilization and metabolic demand during locomotion requires integrated control of the autonomic, cardiovascular, respiratory, and skeletal muscle systems (Coovadia et al., 2020; Barnes and Charkoudian, 2021). From O_2_ in the ambient air to O_2_ delivery to the mitochondria to produce energy (ATP), there are highly coordinated, rapid, and complex adaptive responses (Krogh and Lindhard, 1913; Hughson, 2009; McArdle et al., 2010). Gas exchange in the skeletal muscle must be connected to the pulmonary gas uptake to guarantee energy for muscle contraction and movement (Krogh and Lindhard, 1913); accordingly, O_2_ must be transported from the lungs to the skeletal muscle, otherwise aerobic energy cannot be produced in the mitochondria (McArdle et al., 2010). This O_2_ transport depends on the cardiovascular system (heart and blood vessels) over large distances throughout the body, and local microvascular control and metabolism within skeletal muscle (Poole, 2019).

During physical activity and/or exercise, skeletal muscle O_2_ and ATP demand rises immediately, disrupting physiological homeostasis and generating an “energetic crisis.” To resolve the crisis, rapid and coordinated responses are necessary to adjust the new skeletal muscle O_2_ demand (utilization) to the O_2_ supply (transport and delivery)—thereby re-establishing balance between O_2_ delivery and O_2_ consumption (Laughlin and Joyner, 2003). This matching between O_2_ supply with skeletal muscle metabolic demands depends on the relationship between cardiac output (blood flow), arterial saturation, hemoglobin concentration, vasodilation, O_2_ extraction, arterial and venous O_2_ content, and oxygen consumption (VO_2_) (Hughson, 2009). This way, accelerated mitochondrial ATP turnover (site of O_2_ consumption) during physical activity or exercise must be connected with pulmonary and cardiovascular systems. Therefore, there is a strong association between muscle work, O_2_ delivery, and O_2_ utilization, which is essentially linear during steady-state exercise, except for transitions (Hughson, 2009; Joyner and Casey, 2015). During transitions from rest to exercise, $\dot{V}$ O_2_ is delayed, but the ATP utilization is high. As a result, other ATP sources (anaerobic energy) are needed to sustain muscle work, generating a transient O_2_ deficit until a new steady is reached and aerobic energy production is resumed (Powers et al., 1985).

The dynamic profiles of the cardiovascular, respiratory, and muscular systems during transitions from resting to moderate-intensity constant work rate exercise is known as kinetics analysis (Poole and Jones, 2012; Murias and Paterson, 2015). For VO_2_ kinetics, pulmonary VO_2_ responses present three phases: 1) phase 1 or cardiopulmonary phase, 2) phase 2 or fundamental component, and 3) phase 3 or the steady state of VO_2_ (Murias and Paterson, 2015; Keir et al., 2016). Phase 1 (cardiopulmonary phase) describes the circulatory transient delay between O_2_ utilization from active tissues and adaptation of the pulmonary system. This phase is related to pulmonary blood flow increase, not reflecting increase at the muscular level. Phase 2 (fundamental component) reflects the exponential increase in pulmonary VO_2_ due to increases in pulmonary and muscle blood flow, increased O_2_ extraction in skeletal muscle, and deoxygenated blood return from the active muscles to the pulmonary system. It is described by a mono-exponential response until a steady state is achieved, representing closely the oxidative phosphorylation adjustments in the active muscles. Phase 3 represents the pulmonary VO_2_ steady state where oxygen delivery and oxygen utilization are matched throughout the cardiorespiratory and muscular systems.

Kinetic analysis of VO_2_ provides insight into the integrated mechanisms regulating muscle energetics and oxidative function (Poole and Jones, 2012; Murias and Paterson, 2015). VO_2_ is a standardized and quantified metric for investigating and detecting aerobic fitness and physical activity levels in human subjects (Powers et al., 1985; Beltrame et al., 2017a). Furthermore, pulmonary VO_2_ kinetics provide information about the O_2_ deficit, and how much (amplitude) and how fast or slow the physiological systems adapt during moderate-intensity exercise, which is also related to aerobic fitness (Powers et al., 1985). Faster VO_2_ kinetics for a given metabolic demand results in lower O_2_ deficit, less substrate level phosphorylation and high exercise tolerance. By contrast, slower VO_2_ kinetics results in a higher O_2_ deficit imposing greater challenges for homeostasis and higher exercise intolerance (Poole and Jones, 2012). Therefore, a person with a slower VO_2_ kinetic response and higher O_2_ deficit during exercise indicates an impaired response. Consequently, well-regulated autonomic, cardiovascular, respiratory, and muscular systems allow people to successfully respond to physiological stressors through rapid matching between O_2_ demand and supply (Hughson, 2009; Beltrame et al., 2017b; Beltrame and Hughson, 2017). By contrast, a mismatch between O_2_ delivery and O_2_ utilization may compromise the ability to adjust rapidly to imposed stressors in pathological conditions (Poole and Jones, 2012), and might expose people to life-threatening risks (e.g., physical inactivity, sedentary behavior, frailty, chronic diseases) and hazards (e.g., falls). Impaired O_2_ delivery and O_2_ utilization by the integrated dynamic autonomic, cardiovascular, respiratory, and muscular systems are strongly associated with loss of independence, lower exercise capacity, higher morbidity and mortality rates (McPhee et al., 2016; Booth et al., 2017), increased healthcare utilization, reduced wellbeing, and reduced quality of life (Otones et al., 2020). As a result of such dysfunctions, simple daily activities may become more challenging to be performed, creating a ‘vicious cycle’ of increased physical inactivity, reduced mobility, and poorer health outcomes over time.

Previous research has reported that older adults and people living with chronic disease often experience premature exhaustion, early onset of fatigue, reduced exercise tolerance (Sietsema et al., 1994; Poole and Musch, 2010; Poole et al., 2012; Hirai et al., 2015); collectively, these symptoms can be understood as weakness resulting from impaired regulation of autonomic, cardiovascular, and respiratory systems (Garg et al., 2014; Goulding et al., 2018). Blunted vasodilatory responses during exercise limit blood flow and O_2_ delivery to the active skeletal muscles. The reduced capacity to achieve homeostasis with aging and chronic diseases is quantified as slower VO_2_, heart rate, and cardiac output kinetic responses, with a longer time to reach steady state during rest-to-exercise transitions and at a constant work rate (Sietsema et al., 1994; Meyer et al., 1998; Poole and Musch, 2010; Borghi-Silva et al., 2012; Poole et al., 2012; Hirai et al., 2015). For example, Murias and Paterson’s review (Murias and Paterson, 2015) reported that older adults show slower VO_2_ kinetic responses than healthy young adults, partly due to limitations in O_2_ delivery. Older and young adults performed constant work rate moderate-intensity exercise for 6 min at 90% of their estimated lactate threshold. The authors concluded that slower VO_2_ adaptation in older adults is probably the result of increased metabolic inertia and reduced O_2_ availability. Additionally, Sietsema et al. (Sietsema et al., 1994) showed that patients with chronic heart failure (CHF) had slower VO_2_ kinetic responses as measured by metabolic cart, taking longer to reach steady state than healthy age-matched participants. Participants (CHF and control group) exercised in a cycle ergometer for 6 min at a constant work rate matched by absolute and relative work rate (50% between lactate threshold and maximal exercise). The authors concluded that the slower adaptations in CHF from maximal and submaximal exercise indicate the presence of exercise intolerance in this population. Borghi-Silva et al. (Borghi-Silva et al., 2012) showed that VO_2_ and heart rate kinetics were slowed in patients living with chronic obstructive pulmonary disease (COPD). COPD patients and healthy age and sex-matched controls underwent 6 min of a constant work rate moderate-intensity exercise on a treadmill at 70% of their maximal intensity. The authors concluded that the slower VO_2_ and HR responses in moderate-severe COPD patients are associated with impaired O_2_ delivery and O_2_ utilization during exercise. Thus, patients with COPD experience defects in skeletal muscle oxygenation that reduce exercise capacity independent from their pulmonary impairments (Hussain et al., 2008). In patients with peripheral artery disease (PAD), previous studies showed slower VO_2_ kinetics during treadmill walking at a moderate-intensity constant work rate (Bauer et al., 1999; Barker et al., 2004; Ritti-Dias et al., 2013). The slower VO_2_ kinetics contributed to walking intolerance, differences in muscle carbohydrate oxidation (Barker et al., 2004), altered control of skeletal muscle metabolism (Bauer et al., 1999), and are attributed to both central (cardiac) and peripheral (skeletal muscle) alterations (Ritti-Dias et al., 2013).

The use of NIRS is a well-established and natural adjunct tool for studying the relationship between pulmonary and muscle O_2_ kinetics during exercise (Poole and Jones, 2012; Grassi and Quaresima, 2016; Barstow, 2019). NIRS has been applied during incremental exercise protocols (Ferreira et al., 2007; Spencer et al., 2012; Murias et al., 2013), and can also be used to identify anaerobic threshold (Rao et al., 2012; Reis et al., 2013; Jones et al., 2017) or oxygen dynamic break points (Iannetta et al., 2017). Sperandio et al. (Sperandio et al., 2009) used NIRS in patients with heart failure to demonstrate an “overshoot” in skeletal muscle deoxygenation profile (i.e., deoxy-hemoglobin) with exercise, signifying impaired microvascular oxygen delivery and increased skeletal muscle oxygen extraction. Delorey et al. (DeLorey et al., 2004) tested the effects of age on VO_2_ kinetics and muscle oxygenation adaptations at the onset of moderate-intensity cycling exercise (80% estimated lactate threshold). VO_2_ kinetics was measured breath-by-breath with a metabolic cart, and muscle oxygenation using NIRS. The authors showed that older adults had a slower pulmonary VO_2_ response and longer time to reach steady state than healthy young adults. The slower pulmonary VO_2_ kinetics in older adults was accompanied by reduced skeletal muscle blood flow and O_2_ delivery, and pronounced muscle deoxygenation. Gurd et al. (Gurd et al., 2008) also demonstrated slower VO_2_ kinetics responses and greater deoxyhemoglobin profile in older adults compared with healthy young adults. Machado et al. (Machado et al., 2017) showed that males with metabolic syndrome (MetS) had altered muscle deoxygenation kinetics during incremental exercise. Participants with MetS and age-matched controls performed a progressive and incremental cardiopulmonary exercise test in a cycle ergometer. Ventilatory parameters (VO_2_, VCO_2_, minute ventilation) were measured on a breath-by-breath basis using a metabolic cart and deoxygenation by NIRS. The authors concluded that in MetS, skeletal muscle O_2_ supply-demand matching is impaired, and contributes to reduced aerobic power and exercise capacity. Similarly, McClatchey and others (McClatchey et al., 2017) found an increased deoxyhemoglobin profile in patients with Type 2 diabetes; in contrast with healthy controls, changes in skeletal muscle deoxygenation in patients with diabetes were dissociated from global markers of VO_2_.

Advanced methodologies can be used to further dissect the etiology of reduced oxygen consumption at the muscular level, specifically examining the contributions of impaired mitochondrial function and local oxygen delivery to reduced exercise tolerance. This is important given the limitations of global evaluations of oxygen consumption (i.e., pulmonary VO_2_) to identify localized defects in skeletal muscle O_2_ supply *versus* tissue utilization (Lanza and Nair, 2010). For example, isolated limb exercise is a metabolic challenge with submaximal systemic effects that can interrogate oxygen kinetics within a dedicated peripheral compartment. This technique was applied by Gifford and others (Gifford et al., 2016) comparing knee extension (peripheral) and cycling (systemic) exercise, while using doppler and blood gas measurements of the femoral artery, and *ex vivo* assessment of mitochondrial function from skeletal muscle biopsies. In this study, researchers showed that untrained individuals limit global VO_2_ due to skeletal muscle mitochondrial respiratory capacity despite adequate O_2_ supply, whereas trained individuals experience VO_2_ limitations due to inadequate O_2_ supply despite excess mitochondrial capacity (Gifford et al., 2016). ^31^Phosphorous-magenetic resonance spectroscopy (MRS) can also be used to continuously measure phosphocreatine (PCr) recovery *in vivo* as a marker of mitochondrial oxidative capacity *in vivo*, and variations to inspired fraction of oxygen during exercise can modulate global oxygen delivery; in addition to muscle oxygenation (i.e., balance between delivery and utilization) that is evaluated with NIRS, arterial doppler can directly quantify blood flow and convective oxygen delivery. These multi-modal protocols have generated important insights regarding skeletal muscle exercise physiology in healthy and disease conditions. Haselar and others (Haseler et al., 1999) showed that during submaximal plantar flexion exercises in exercise-trained adults, PCr recovery time constants were dependent on inspired fraction of oxygen, and thus oxygen availability. Combination of NIRS and PCr can be used to estimate of skeletal muscle capillary blood flow (Harper et al., 2008; Layec et al., 2013). Using this technique, in addition to doppler blood flow of the popliteal artery, Layec and others demonstrated capillary blood flow and PCr recovery is preserved in COPD patients, despite delayed whole-limb blood flow recovery following plantar flexion exercises; the authors concluded that increased fatigue in COPD patients may be related to inefficient distribution of blood flow throughout the leg, as opposed to intrinsic mitochondrial dysfunction of exercising muscle. Similarly, Hart and others (Hart et al., 2018) demonstrated preserved mitochondrial capacity using MRS and *in vitro* techniques in patients with peripheral arterial disease, and markedly abnormal bulk blood flow and oxygen delivery as assessed by doppler, and abnormal deoxygenation profiles as assessed by NIRS; together, these data clearly suggest blood flow limitations, as opposed to mitochondrial dysfunction, as the primary defect in this patient population. Pilotto and others (Pilotto et al., 2022) used NIRS applied to the vastus lateralis in association with incremental exercise and arterial occlusion to determine muscle O_2_ diffusion constants, and showed that they were inversely correlated with capillary density on skeletal muscle biopsies. Therefore, as outlined by these selected studies, additional testing with refined protocol design can potentially be used to interrogate mechanisms of skeletal muscle dysfunction in ICU-AW beyond whole-body oxygen kinetics.

**FIGURE 2:**
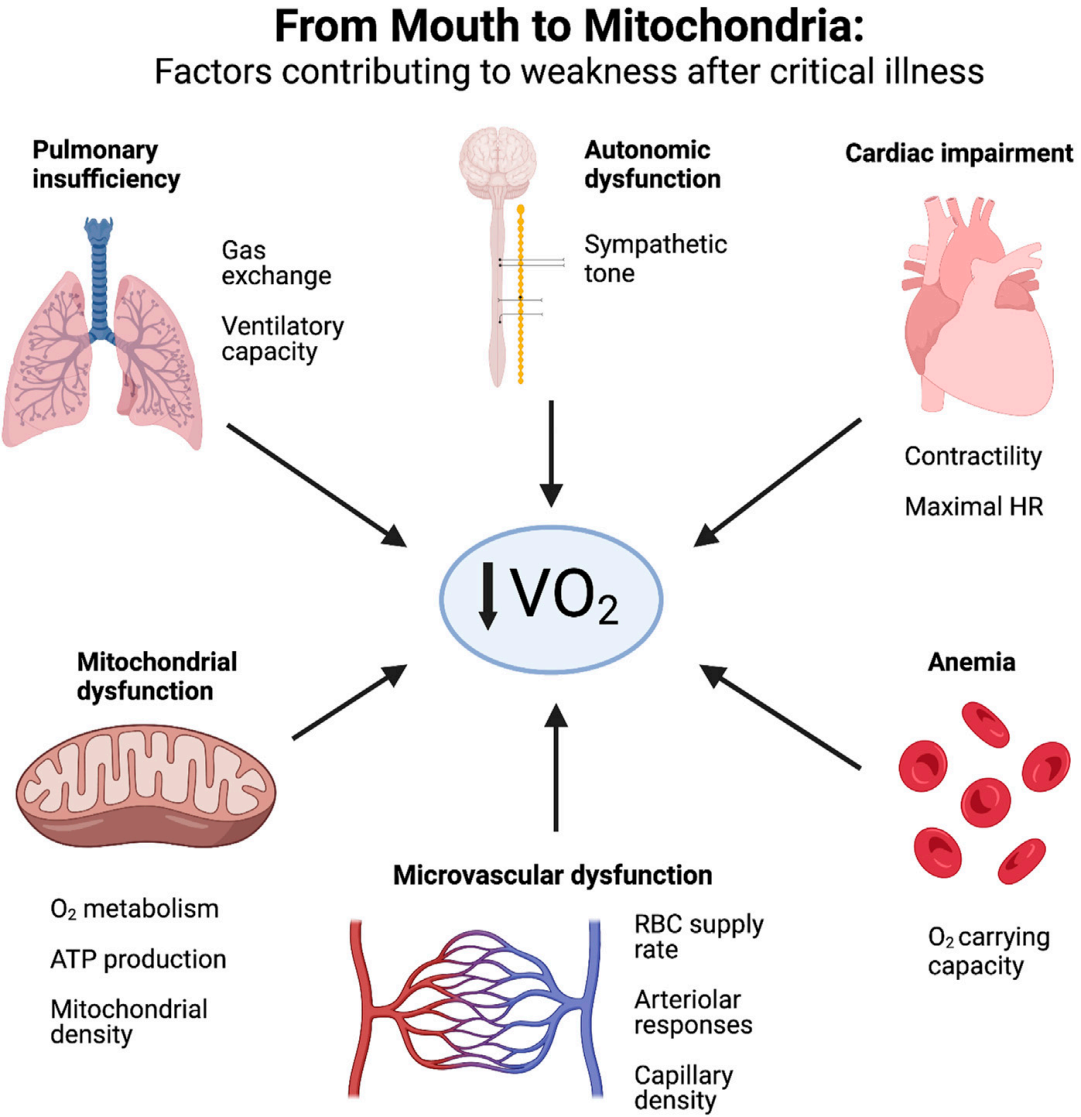
Factors contributing to weakness after critical illness. ICU survivors experience physiological defects along the entire oxygen delivery pathway–from mouth to mitochondria—that restrict oxygen consumption (VO_2_) and manifest as reduced exercise capacity and weakness. *Created with* *BioRender.com* .

Overall, it is very likely that survivors of critical illness suffer from defects throughout the O_2_ delivery pathway–from mouth to mitochondria (Figure 2). A systematic evaluation of each physiological domain may provide further insight into the mechanisms of ICU-AW and impaired exercise capacity. Starting in the respiratory system, residual parenchymal lung damage (Ekbom et al., 2021), as well as ventilatory and diaphragmatic muscle atrophy and dysfunction (Goligher et al., 2018) will limit oxygen uptake and CO_2_ removal. Cardiac dysfunction would manifest as impaired contractility and chronotropy, limiting maximal cardiac output and global oxygen delivery. Anemia is an almost universal finding in critical illness (Hayden et al., 2012; Warner et al., 2020), and if persistent in the recovery phase after critical illness, imposes limitations to O_2_ carrying capacity. Autonomic dysfunction occurs frequently during the acute phase of critical illness due to stress, inflammation and exposure to exogenous catecholamines; inability to restore homeostasis between sympathetic and parasympathetic systems would impair the coordinated hyperemic responses of skeletal muscle blood flow during exercise (Joyner and Casey, 2015). As outlined above, microvascular defects in skeletal muscle are well-described in critical illness and would manifest as both conductive and diffusive limitations to O_2_ delivery. Lastly, mitochondrial dysfunction in skeletal muscle is a cardinal feature of ICU-AW (Friedrich et al., 2015; Lad et al., 2020), and leads to impaired ATP production at the terminal site of O_2_ metabolism and reduced skeletal muscle contractile ability; skeletal muscle biopsies from critically ill patients suggest depletion of mitochondrial complex III and IV concentrations (Jiroutková et al., 2015), as well as decreased citrate synthase activity (Carré et al., 2010) and reduced mitochondrial density (Fredriksson et al., 2006). Thus, when the totality of this O_2_ delivery pathway is considered for ICU survivors, a plausible hypothesis becomes evident that ICU-AW extends well beyond intracellular abnormalities in the myocyte. We suggest that objective and perceived weakness after critical illness represents a physiological failure of O_2_ supply-demand matching - both globally throughout the body and locally within skeletal muscle. This framework represents a paradigm shift in ICU-AW, and enhances the current perspective focused predominantly on skeletal muscle biology.

## Exercise testing for evaluating ICU-AW–New research and rehabilitation opportunities

Recently, there has been growing attention for using established methodologies from exercise physiology in the evaluation of ICU-AW, although this field is still in its infancy. The benefit to this approach is that it provides objective quantitative data for ICU survivors, which may enable improved follow-up and enhanced ability to prescribe personalized rehabilitation strategies (Molinger et al., 2020; French et al., 2022). These types of measurements may also be important for identifying novel therapeutic targets or treatment strategies.

Cardiopulmonary exercise testing (CPET) is an advanced diagnostic technique used clinically for numerous patients with chronic cardiorespiratory diseases (Radtke et al., 2019), and has also been used extensively in the field of exercise science to study aerobic and anaerobic physiology in athletes and healthy adults (Mazaheri et al., 2021). The function of CPET is to delineate which component of a patient’s physiology (heart, lung, other) is most significantly limiting exercise capacity and function. CPET carries a high prognostic value for patients with COPD or heart failure (Nadruz et al., 2017; Lala et al., 2021; Ewert et al., 2022) and can also be used for pre-operative assessment (Levett et al., 2018) and before transplantation (Dudley and El-Chemaly, 2012; Corrà et al., 2018). However, the use of CPET to study ICU-AW has been under-explored despite robust academic and practical potential within this patient population. CPET is safe in frail outpatient populations, and has even been shown to be feasible during ICU admission (Sommers et al., 2019). Almost 20 years ago, Ong and others evaluated 44 patients with acute respiratory distress syndrome 3 months after hospital discharge using pulmonary function and CPET (Ong et al., 2004). They found that 41% of survivors had reduced exercise capacity, as quantified by VO_2peak_, below the lower limit of normal range for age. Importantly, they observed that these functional limitations were not entirely explained by ventilatory impairments, which were found to be only mild. These findings have been corroborated by a series of recent studies. In an expansive cohort of 433 general ICU survivors, Van Aerde and others (Van Aerde et al., 2021) found a significant proportion of patients (37%) had impaired exercise capacity on CPET up to 5 years after ICU discharge, and that muscular limitations frequently (60%) contributed to this observation. However, the description of muscular limitation in this study was signified only by the absence of cardiac (i.e., heart rate) or respiratory (i.e., ventilatory or gas exchange) abnormalities during exercise testing. Further detailed testing was undertaken by Mart and others (Mart et al., 2022) who utilized both standard CPET parameters as well as VO_2_-work rate slope and VO_2_ recovery half-time as markers of oxygen consumption. In 14 participants at 6 months after ICU discharge, 79% demonstrated reduced exercise capacity (VO_2peak_), and abnormal slope and recovery times. They concluded that skeletal muscle oxygen utilization may contribute to impaired exercise capacity, potentially attributed to skeletal muscle mitochondrial function. Joris et al. (Joris et al., 2021) evaluated exercise fitness with CPET and a metabolic cart in 14 survivors of COVID-19 at 3 months and 6 months after ICU discharge. They found reduced peak $\dot{V}$ O_2_ at both time points (80% and 81%, %predicted) as well as reduced maximal workload (67%and and 63%, %predicted). Similar to previous studies, this was not attributed to cardiac or respiratory abnormalities. A hypermetabolic state with increased protein catabolism was observed at 3 months after discharge, as evidenced by a respiratory exchange ratio less than 1. Prolonged half-time of VO_2_ recovery after exercise and reduced metabolic efficiency (workload/peak VO_2_) suggest impaired oxygen extraction and utilization at the level of skeletal muscle–which they attributed to either microvascular or mitochondrial dysfunction; as discussed in the above, determinations of mitochondrial dysfunction may be better evaluated with isolated limb exercises and MRS. Longobardi and others (Longobardi et al., 2022) further evaluated 35 patients with COVID-19 recently discharged from ICU (mean 5 months) as well as age and sex matched controls. They used a hybrid protocol of constant workload and ramped exercise CPET to evaluate peak VO_2_ as well as on/off kinetics. In this study, COVID-19 patients demonstrated greater oxygen deficit, slowed on/off kinetics, abnormal chronotropic heart rate response–all suggesting dramatic impairments in cardiorespiratory fitness despite similar ventilatory threshold to controls.

Together, these recent studies demonstrate the increasing role of CPET for evaluating fitness in ICU survivors, providing quantifiable data to categorize and track recovery, and elucidate mechanisms of disease. Although differences may exist between COVID-19 survivors and general ICU patients, it becomes clear from these studies that residual weakness may reflect abnormalities with oxygen supply throughout the cardiovascular system in addition to intrinsic muscular dysfunction. Notably, direct interrogation of oxygen delivery and utilization at the site of skeletal muscle in ICU survivors is still lacking. As described in previous sections, use of NIRS, isolated limb exercises, and other advanced methodologies during exercise protocols may provide further mechanistic physiological information for ICU-AW by measuring skeletal muscle oxygenation, oxygen delivery and utilization–together, these are natural complements for global markers of exercise capacity that are evaluated with CPET.

## Conclusion

When considering the importance of oxygen delivery and utilization to normal muscle function, and the direct contact between microvascular networks and surrounding muscle, the contribution of the cardiovascular and microvascular systems to the pathogenesis of ICU-acquired weakness is likely under-recognized. There are plausible cellular pathways that underpin crosstalk between endothelial cells and myocytes that may drive skeletal muscle atrophy and limit capacity for regeneration. Persistent microvascular dysfunction after critical illness may cause maladaptive changes in skeletal muscle related to impaired regulation of blood flow and subtrate delivery. Physiological defects throughout the entire oxygen delivery pathway may result in reduced exercise capacity in ICU survivors, and should be considered in addition to pathological changes in the muscle. Future research should focus on elucidating these biological and physiological mechanisms, and incorporating standardized and quantitative exercise testing during post-ICU follow-up.
